# A Comparative Study of Fixed-Dose Dexmedetomidine and Propofol Infusions on Intraoperative Desflurane Consumption During Bispectral Index-Guided Laparoscopic Surgeries: A Randomized Controlled Study

**DOI:** 10.7759/cureus.62479

**Published:** 2024-06-16

**Authors:** Kishore Machani RamaMurthy, Mummaka Harshavardhan, Viren B Attarde, Sujithareddy Karri

**Affiliations:** 1 Anaesthesia and Critical Care Medicine, KEM (King Edward Memorial) Hospital and Research Centre, Pune, IND; 2 Anaesthesia and Critical Care Medicine, Dr. D. Y. Patil Medical College, Hospital & Research Centre, Pune, IND; 3 Cardiac Anaesthesiology, Star Hospital, Hyderabad, IND

**Keywords:** laparoscopic surgeries, desflurane, general anaesthesia, propofol, dexmedetomidine

## Abstract

Background

Desflurane is an excellent but expensive volatile anesthetic agent. Dexmedetomidine and propofol may decrease intraoperative desflurane consumption. This study aimed to compare the desflurane-sparing effect of dexmedetomidine and propofol in patients undergoing laparoscopic surgeries under bispectral index (BIS)-guided general anesthesia (GA).

Methods

Sixty-two adult patients, ASA (American Society of Anesthesiologists) physical status I or II, of either sex, aged between 18 and 60 years, were randomly allocated into group D or group P. Only group D patients received an intravenous (IV) bolus of dexmedetomidine (1 mcg/kg) over 15 minutes before induction. In both groups, GA was induced following the standard protocol with propofol infusion (0.5 mg/kg/min) until the BIS value dropped below 60. For maintenance, group D and group P patients received IV dexmedetomidine infusion (0.5 mcg/kg/h) and propofol infusion (50 mcg/kg/min), respectively. In both groups, desflurane dial concentration was adjusted between 3 and 8% to maintain the BIS within the range of 45-55. An hourly bolus of IV fentanyl (0.5 mcg/kg) and a half-hourly bolus of IV vecuronium (0.02 mg/kg) were administered. The total amount of desflurane consumed, duration of pneumoperitoneum, extra aliquots of propofol used during maintenance, number of boluses of IV atropine, fentanyl, and esmolol, time to attain Ramsay Sedation Score of 2 after extubation, time to first postoperative analgesic request at Numerical Rating Scale (NRS) score ≥ 4, time to reach a Modified Aldrete Score of ≥9, and incidence of any side effects were recorded. All the data were analyzed and compared using appropriate statistical tests, and a p-value of <0.05 was considered significant.

Results

The final data analysis was performed on 60 patients. The mean desflurane consumption was clinically higher in group P patients than in group D, but the difference was statistically insignificant (p-value > 0.05). The mean induction dose of propofol was significantly less in group D than in group P (p-value < 0.05). After extubation, the difference in time to the first analgesic request (NRS ≥ 4) between the groups was statistically significant (p-value < 0.05). Group D patients had a residual intraoperative analgesic effect.

Conclusion

The effects of dexmedetomidine and propofol infusions on desflurane consumption in laparoscopic surgeries are comparable, with minimal effects on intraoperative hemodynamics and postoperative recovery profiles.

## Introduction

Laparoscopic surgeries are associated with reduced postoperative pain and hospital stay, resulting in better patient acceptance [1]. Rapid and smooth postoperative recovery of protective reflexes and psychomotor function is essential for daycare laparoscopic surgeries. Hence, appropriate anesthetic agents or techniques are necessary to maintain adequate depth and facilitate early recovery. The inhalational agent desflurane offers easy titratability and enables a faster emergence due to its favorable pharmacokinetic profile [2]. However, reducing desflurane consumption has been advocated because of higher costs and greenhouse effects [3].

The intravenous (IV) anesthetic agent propofol allows rapid induction and prompt recovery from general anesthesia (GA). A combination of propofol target-controlled infusion and low-concentration desflurane for the maintenance of GA was reported to provide stable hemodynamics in patients undergoing laparoscopic cholecystectomy [4].

Dexmedetomidine provides dose-dependent sedation, anxiolysis, and analgesia without respiratory depression. Continuous IV dexmedetomidine infusion significantly reduces desflurane consumption with attenuation of the hemodynamic response to intubation, pneumoperitoneum, and extubation [5].

To date, the effects of fixed-dose IV dexmedetomidine and propofol infusion on desflurane consumption have not been compared. We investigated and compared the dose-sparing effects of dexmedetomidine and propofol on desflurane consumption using bispectral index (BIS)-guided GA in patients undergoing laparoscopic surgeries. We also assessed the sedation score after extubation, time for the first analgesic request, and discharge time from the recovery room.

## Materials and methods

**Figure 1:**
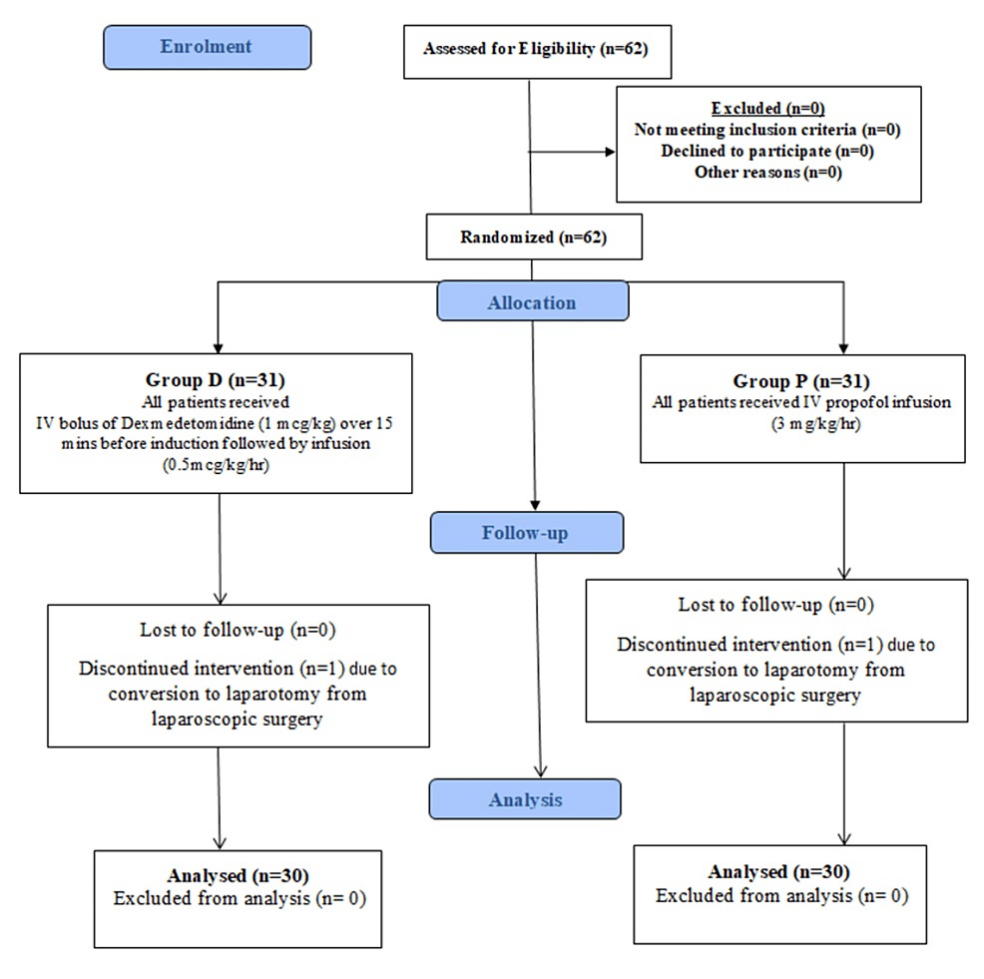
Consort diagram

This study was conducted in the surgical gastroenterology and surgical oncology operation theaters of a tertiary care center, Sri Venkateswara Institute of Medical Sciences, from March 2019 to February 2020. Prior ethical permission was obtained from the Institutional Ethics Committee, Tirupati (AS/11/IEC/SVIMS/2017, IEC No. 860). This study was also registered in the Clinical Trials Registry India (CTRI/2019/08/020485). This prospective, randomized, controlled, single-blind study included 60 American Society of Anesthesiologists (ASA) physical status I and II patients of either sex, aged 18-60 years, weighing 50-80 kg, and undergoing various elective laparoscopic surgeries under GA. The consort diagram indicating enrollment and progress is shown in Figure 1.

Patients with a history of significant systemic disease, known hypersensitivity to the study drugs, alcohol or drug abuse, pregnant women, and lactating mothers were excluded from the study. Patients who required mechanical ventilation postoperatively and those who did not cooperate or refused to participate in the study were also excluded.

A pre-anesthetic assessment was done a day before the surgery, which involved a thorough patient evaluation. All patients were informed about the study protocol and the Numerical Rating Scale (NRS) [6] for the assessment of postoperative pain. Written and informed consent was obtained from each patient. Demographic data such as age, sex, weight, body mass index (BMI), and ASA grade [7] were noted. All study participants received oral alprazolam 0.25 mg and pantoprazole 40 mg the night before surgery.

Patients were randomized on the day of the surgery to one of the two groups, D or P, using a computer-generated random number table and a sealed opaque envelope technique. An 18G intravenous (IV) cannula was secured in the operating room, and IV fluid was started. ASA standard monitors were attached, including electrocardiography, non-invasive blood pressure, pulse oximetry, end-tidal carbon dioxide (EtCO_2_), and BIS. The baseline values of heart rate (HR), systolic blood pressure (SBP), diastolic blood pressure (DBP), mean arterial pressure (MAP), and oxygen saturation (SpO_2_) were recorded.

All participants received IV midazolam (0.03 mg/kg) and fentanyl (2 mcg/kg) and were pre-oxygenated with 100% oxygen for three minutes. Group D patients received an IV bolus of dexmedetomidine (1 mcg/kg) over 15 minutes before induction. GA was induced with IV propofol infusion (0.5 mg/kg/min) until the BIS value dropped below 60. After checking ventilation, IV vecuronium (0.1 mg/kg) was administered to facilitate endotracheal intubation with an appropriate-sized cuffed endotracheal tube (ETT). After confirming the position of the ETT, the lungs were ventilated with 50% O_2_ in air, maintaining a tidal volume of 6-8 mL/kg, respiratory rate of 8-12 per minute, and a PEEP of 5 cm of H_2_O. Minute ventilation was adjusted to maintain normocapnia (EtCO_2_ between 30 and 40 mm Hg). During laparoscopy, intra-abdominal pressure was kept between 12 and 14 mm Hg.

GA was maintained as per random group allocation: groups D and P received IV dexmedetomidine infusion (0.5 mcg/kg/h) and IV propofol infusion (50 mcg/kg/min), respectively. In both groups, desflurane dial concentration was adjusted between 3% and 8% to maintain the BIS within the range of 45-55. An hourly bolus of IV fentanyl (0.5 mcg/kg) and a half-hourly bolus of IV vecuronium (0.02 mg/kg) were administered. Hemodynamic variables were monitored throughout the procedure.

The last relaxant dose was given about 30 minutes before the anticipated end of surgery. All anesthetic agents were discontinued before the suturing of laparoscopic ports, and IV ondansetron (0.1 mg/kg) was given. The neuromuscular block was reversed with IV neostigmine (0.05 mg/kg) and glycopyrrolate (0.01 mg/kg), and the trachea was extubated. The following recordings were noted before shifting the patient from the operation theater: the total amount of desflurane consumed (mL/h) (desflurane consumption calculation) [5], duration of pneumoperitoneum, extra aliquots of propofol used during maintenance, number of boluses of IV atropine, fentanyl, and esmolol.

In the post-anesthesia care unit, hemodynamic monitoring was continued, and fluids were administered as per the deficit. The following time points were recorded: time to attain Ramsay Sedation Score [8] of 2 after extubation (in minutes), time from extubation to the first postoperative analgesic request at NRS score ≥ 4 (in minutes). If the NRS score was ≥4, IV paracetamol (15 mg/kg), followed by IV tramadol (1.5 mg/kg), was given. The time needed for the patients in both groups to reach a Modified Aldrete Score [9] of 9 (minutes) was also recorded.

Hypertension and hypotension were defined as a rise and drop in SBP by more than 20%, respectively [10]. Tachycardia and bradycardia were described as heart rates >100 and <50 beats per minute, respectively [11]. If hypertension and/or tachycardia were observed with BIS more than the recommended range (45-55), then desflurane dial concentration was increased to a maximum of 8%. If the rise in hemodynamics persisted, 10 mg aliquots of propofol were given up to a maximum of 1 mg/kg. In case of persistent hypertension and tachycardia with BIS within the recommended range, IV esmolol 0.1 mg/kg bolus was given. IV nitroglycerin infusion (25-100 mcg/min) was initiated if only hypertension persisted. Hypotension with tachycardia was initially treated with 200 mL of fluid followed by an IV bolus of phenylephrine (50 mcg). The incidence of bradycardia with or without hypotension was treated with an IV bolus of atropine (0.6 mg/kg). Isolated hypotension was treated with an IV bolus of ephedrine (0.06 mg/kg).

The sample size was estimated from the study by Khafagy et al. [5]. The mean dose of desflurane consumed was 22.47 ± 1.71 mL/hour in the placebo (saline) group compared to 16.47 ± 1.65 mL/h in the dexmedetomidine group. The sample size was calculated considering a probability of 0.05 and 80% power with an anticipated effect size (Cohen’s d = 0.8). The required sample size was 50 participants, or 25 participants in each group. Considering an attrition rate of 20%, 30 patients in each group were included in the study. However, we recruited 62 participants, and there were no dropouts. Two patients were excluded from our study due to conversion to laparotomy from laparoscopic surgery. The final data analysis was performed on 60 patients (Group P = 30 patients, Group D = 30 patients).

All collected data were represented in an Excel chart (Microsoft Corporation, Redmond, Washington) and double-checked for clerical errors. Data were analyzed using standard statistical software IBM SPSS Statistics for Windows, Version 24 (Released 2016; IBM Corp., Armonk, New York). The variability in data obtained was expressed either as median with range for nonparametric data or mean with standard deviation for normally distributed data. Continuous data such as age, height, weight, and BMI were analyzed. Categorical data such as gender and ASA grading were analyzed using the chi-square test or Fisher’s exact test, as appropriate. Anesthetic adverse events were analyzed using the chi-square test or Fisher’s exact test. Extra aliquots of propofol required during maintenance and the number of boluses of IV fentanyl, atropine, esmolol, and phenylephrine were analyzed with the chi-square test or Fisher’s exact test, as appropriate. A p-value of <0.05 was considered statistically significant.

## Results

We recruited 62 participants, and there were no dropouts. Two patients were excluded from our study due to conversion to laparotomy from laparoscopic surgery. Thus, the final data analysis was performed on 60 patients (Group P = 30 patients, Group D = 30 patients). Both groups were comparable in demographic profiles (age, gender, ASA grade, weight, height, BMI) (Table 1) (p > 0.05).

**Table 1:** Demographic Profile (mean ± SD) ASA PS: American Society of Anesthesiologists physical status, BMI: body mass index.

Parameters	Group D (n=30)	Group P (n=30)	p-Value
Age (years)	44.67±10.35	42.20±11.83	0.395
Gender (M/F)	15/15	17/13	0.605
ASA PS (I/II)	15/15	16/14	0.796
Weight (kg)	61.86±10.20	63.90±12.60	0.495
Height (cm)	157.36±9.79	159.40±8.97	0.405
BMI (kg/m^2^)	26.32±4.11	26.33±3.96	0.994

Table 2 shows the consumption of desflurane among the study participants in both groups. The mean consumption was higher in group P patients than in group D. However, the difference was statistically insignificant (p > 0.05). There was no statistically significant difference between the groups in the total duration of pneumoperitoneum (p > 0.05). The mean induction dose of propofol was significantly less in the dexmedetomidine group compared to the propofol group (p < 0.05). There was no statistically significant difference between the two groups regarding the requirement for extra propofol or fentanyl boluses (p > 0.05) (Table 2).

**Table 2:** Intraoperative Profile (Mean ± SD)

Parameters	Group D (n=30)	Group P (n=30)	p-Value
Desflurane consumption (mL/hour), median + interquartile range	10.335+7.18	13.335+11.13	0.209
Duration of pneumoperitoneum (min), mean ± SD	57.76 ± 24.82	71.6 ± 32.95	0.07
Induction dose of propofol (mg)	66.00±14.04	108±24.50	0.00
Extra aliquots of propofol consumed			
2	1	1	0.735
3	3	2	
5	1	0	
No. of boluses of fentanyl			
1	11	18	0.05
2	1	3	

After extubation, the difference in time to the first analgesic request (NRS ≥ 4) between the groups was statistically significant (Table 3) (p < 0.05). Group D patients had a residual intraoperative analgesic effect. There was no statistically significant difference between the groups in the attainment of an RSS > 2 and the time to attain a Modified Aldrete score of ≥ 9 following extubation (p > 0.05).

**Table 3:** Postoperative Profile (After Extubation) (Mean ± SD)

Parameters	Group D (n=30)	Group P (n=30)	p-Value
Time to first analgesic request (min)	23.43±9.88	11.30±5.82	0.00
Time to achieve a Ramsay sedation score of 2 (min)	10.56±2.52	9.96±2.07	0.319
Time to reach Modified Aldrete score of ≥ 9	9.43±1.47	9.83±2.42	0.443

The incidence of adverse effects is compared in Table 4. There was a higher incidence of hypertension in group P than in group D, which was statistically significant (p < 0.05).

**Table 4:** Adverse Effects

Parameters	No. of Episodes	Group D (n=30)	Group P (n=30)	p-Value
Hypotension	1	6	3	0.350
	2	0	1	
Hypertension	1	11	13	0.008
	2	0	7	
	3	0	1	
Tachycardia	1	8	11	0.070
	2	2	7	
Bradycardia	1	2	1	0.550

## Discussion

The findings of the present randomized controlled trial revealed no significant differences in the consumption of desflurane between the two groups. However, group D patients had an hourly 22.5% lesser desflurane consumption than group P. The mean induction dose of propofol was significantly less in patients who received dexmedetomidine infusion. There was a significant delay in the first analgesic request in group D. The two groups were comparable in requirements for extra aliquots of propofol, IV fentanyl bolus, the time required to achieve an RSS of 2, and a Modified Aldrete score of ≥ 9. The incidence of adverse events was similar, except episodes of hypertension were more in group P.

Numerous studies have suggested that dexmedetomidine has an anesthetic-sparing property [12-16]. The hypnotic and supraspinal analgesic effects of dexmedetomidine occur due to the suppression of neuronal firing in the locus coeruleus and inhibition of norepinephrine release [17]. In most of the published literature, dexmedetomidine was administered as an IV loading dose in a short time (1 μg/kg over 10 minutes) and maintenance (0.2-0.7 μg/kg/h) [18-20].

Khafagy et al. [5] also observed a 27% lower consumption of desflurane with dexmedetomidine during BIS-guided GA for elective laparoscopic cholecystectomy.

We observed that there is no significant difference in reducing the requirement of desflurane with a fixed-dose IV infusion of dexmedetomidine (0.5 mcg/kg/hr) and propofol (50 mcg/kg/min). Our result could be affected by administering the loading dose over 15 minutes and the anesthetic-sparing action of fentanyl during maintenance. Moreover, the dose of our study drugs might not have been equianesthetic.

Keniya et al. and Harsoor et al. reported a significant reduction in the hourly requirement of isoflurane and sevoflurane, respectively [21, 22].

In our study, dexmedetomidine also helped to reduce the propofol requirement during induction without any significant hemodynamic alteration. Intraoperative vitals were relatively stable with dexmedetomidine compared to the fixed-dose IV infusion of propofol.

Vora et al. [23] administered a dexmedetomidine bolus (1 mcg/kg/h), followed by continuous infusion (0.5 mcg/kg/h), and observed significantly lower intraoperative HR and blood pressure. Three patients had bradycardia in the postoperative period.

Khafagy et al. [5] and Keniya et al. [21] reported symptomatic bradycardia in one and two patients, respectively, which required pharmacologic intervention.

In our study, only two patients in the dexmedetomidine group and one in the propofol group had asymptomatic bradycardia, which was self-limiting. We did not use the loading dose to avoid symptomatic bradycardia or arrhythmia.

Bhutia and Rai [24] also did not use a loading dose but noticed significant hypotension in one and two patients with propofol and dexmedetomidine, respectively. Our findings are well-matched with the observations of Shah et al. [25]. There was no significant difference in HR between the propofol and dexmedetomidine groups. Hence, propofol and dexmedetomidine were equally effective in attenuating the HR increase due to the sympathetic response to pneumoperitoneum.

Patients in the propofol group received significantly higher boluses of IV esmolol, which was used to treat more than a 20% increase in HR and SBP (p = 0.003). The higher usage of esmolol in the propofol group was explained by the higher incidence of hypertensive episodes in the propofol group compared to the dexmedetomidine group (21 vs. 11 episodes).

Both propofol and dexmedetomidine produce sedation through different mechanisms. Dexmedetomidine induces sleep by hyperpolarizing noradrenergic locus ceruleus neurons [14], whereas propofol acts through a gamma-aminobutyric acid (GABA) agonistic effect. We observed no difference between the two groups with a fixed dose of the study drug infusion in achieving a Ramsay sedation score of 2 and a Modified Aldrete Score of 9.

On the contrary, Bhutia et al. [24] observed that the patients in the dexmedetomidine group required more time to achieve a Ramsay sedation score of 2 post-extubation (p = 0.02). The difference in their result from our findings could be due to their use of a mixture of oxygen and nitrous oxide (50:50), whereas we used 50% oxygen in the air. Nitrous oxide might have further potentiated the sedative and analgesic effects of dexmedetomidine.

Chattopadhyay et al. [26] observed that patients in the dexmedetomidine group were more sedated than those in the propofol group, but without any impairment of ventilation or recovery. This difference with our study could be because all our study participants received an additional 0.5 mcg/kg/h of fentanyl during the maintenance period, which might have affected the recovery of patients equally in both groups.

The time to the first analgesic request was significantly longer in the dexmedetomidine group than in the propofol group. This was because dexmedetomidine infusion had a specific analgesic effect and provided visceral pain relief [27]. In a comparative study between dexmedetomidine and propofol for short surgical procedures, Harinath et al. [28] found no difference in the time taken to achieve an Aldrete score of 10. Our study results concur with the findings mentioned above. Additionally, both studies involved shorter surgical durations, which might have diminished the sedative effects of dexmedetomidine.

Our study has a few limitations. We did not measure the end-tidal concentration of desflurane and serum concentrations of dexmedetomidine and propofol during maintenance. A multicentric study with a large sample size and its feasibility in ASA III and IV patients need to be explored. Further studies can also be planned using target-controlled propofol infusion.

Finally, with fixed-dose dexmedetomidine and propofol infusions, additive anesthetic effects were delivered along with intraoperative desflurane.

## Conclusions

Our study concludes that the effects of dexmedetomidine and propofol infusions on desflurane consumption during BIS-guided laparoscopic surgeries are comparable. Both agents have minimal effects on intraoperative hemodynamics and the postoperative recovery profile.
